# Systems Are Overstretched from the COVID-19 Pandemic: An Interpretive Description of Disabled People’s Access to Healthcare and Disability Support in New Zealand

**DOI:** 10.3390/healthcare12030387

**Published:** 2024-02-02

**Authors:** Solmaz Nazari Orakani, Tara N. Officer, Gretchen Good, Karen McBride-Henry

**Affiliations:** 1 School of Nursing, Midwifery and Health Practice, Wellington Faculty of Health, Victoria University of Wellington, Wellington 6012, New Zealand; tara.officer@vuw.ac.nz (T.N.O.); karen.mcbride-henry@vuw.ac.nz (K.M.-H.); 2 School of Health Sciences, Massey University, Palmerston North 4442, New Zealand; G.A.Good@massey.ac.nz

**Keywords:** healthcare, disability support services, COVID-19 pandemic, disabled people

## Abstract

The COVID-19 pandemic disrupted healthcare and support services, creating challenges for disabled people. New Zealand implemented a range of policies to prevent and limit viral transmission of COVID-19. This study investigates disabled people’s experiences accessing healthcare and disability support services during the COVID-19 pandemic, and based on this analysis, the implications of public health policy decisions on disabled people’s experiences during the pandemic in New Zealand are explicated. A qualitative design underpinned by interpretive description methodology guided this study. A total of 64 disabled people or parents of disabled children participated in semi-structured interviews. The team of health services and disability researchers then engaged in an iterative thematic approach to analysis, which led to three key themes: (1) protective personal factors, which assisted disabled people to access healthcare and support services, (2) immediate pandemic policy impacts, including policy and legislative changes, which created additional access barriers for disabled people, and (3) exacerbating factors, including compounding vulnerabilities, overstretched systems, and the impact of the vaccine mandate, which worsened the already limited access to healthcare and disability services for disabled people. The pandemic overwhelmed an already stretched healthcare and disability support system, resulting in service disruptions with negative consequences for disabled people’s health and wellbeing. Future policy development needs to be disability-centred in its inclusion of people with lived experience and consideration of the support needs of disabled populations. A first step in this process could include pandemic planning and policy co-design to ensure a continuum of healthcare services and support availability for individuals when services are disrupted. In addition, access to formal and informal support for disabled people should be recognised as a fundamental human right when accessing healthcare and disability support services.

## 1. Introduction

Disabled people constitute 11% of the global population [1] and 24% of New Zealand’s population [2]. Despite accessing healthcare at twice the rate of the non-disabled population [3,4], disabled people are more likely to experience health inequities [5]. Additionally, due to a systemic lack of preparation and planning around healthcare access, disabled people also experience worse outcomes in disasters [6]. This is particularly evident during the COVID-19 pandemic, where adverse health outcomes were exacerbated for disabled people [3,7,8,9]. While the pandemic might be considered “over” in some respects, its impact is still being felt by the disabled community, and indeed, some of the ongoing effects (such as long COVID-19 and a severe shortage of support workers) are just emerging.

### 1.1. The New Zealand Experience

New Zealand’s health system is primarily publicly funded, with free hospital services and co-payments for primary care services and, until recently, co-payments for medicines prescribed in the public hospital and primary care space [4]. There are a wide range of government-funded disability supports and services to support disabled people in their everyday life, maintaining their independence and connecting them with the wider community. The recently created Ministry of Disabled People administers these services through disability service providers. In the early stages of the pandemic in New Zealand, the Government enacted the COVID-19 Public Health Response Act 2020, which provided the legal framework for a range of policies aimed at preventing and limiting the risk of COVID-19 outbreak [10]. Consequently, several ‘shelter in place’ orders and ongoing restrictions, such as mask-wearing, and bans on driving except for essential business, were imposed to eliminate COVID-19. Compared to many countries, New Zealand’s initial pandemic response was amongst the most stringent, likely resulting in reduced mortality rates. However, the ongoing restrictions also significantly disrupted healthcare and support services, resulting in challenges for disabled people. For example, staff shortages were exacerbated by infection control measures, staff illness, and mandatory self-isolation periods, resulting in in-home support services being scaled back, disrupted, or even suspended, and many community services, such as day programmes and respite care were also negatively impacted [11,12,13].

### 1.2. The Problem Statement

Early in the pandemic, there were several calls to address specific pandemic challenges for disabled people (see, for example, [14,15]). However, while there is extensive research into the impact of quarantine restrictions globally, including in relation to wellbeing and health services access; there is significantly less research into the implication of these restrictions on disabled populations. In New Zealand, the published evidence suggests that society created additional barriers for disabled people who faced significant difficulties in accessing healthcare and support services [12]. In turn, internationally, there is growing concern around continued perpetuated healthcare and disability support access inequities for disabled people [1]. These inequities are thought to lead to poorer health outcomes and added disability [16]. Significantly, there remains a gap in the literature exploring New Zealand-based literature on the experiences of disabled people and their carers in accessing healthcare and disability support services over the pandemic. Additionally, very little is known about the implications of policy decisions on disabled people’s experiences during the pandemic in New Zealand; this study aims to address this gap by exploring the impacts of the pandemic-related policy and practice on disabled people’s access to healthcare and disability support services.

## 2. Method

This study was guided by Thorne’s [17] Interpretive Description methodology; this methodology enables researchers to develop translational findings that improve clinicians’ ability to respond to what is happening within clinical practice [17]. Interpretive Description offers an accessible and theoretically flexible approach to analysing data. It is particularly helpful when considering explorative novel topics, where the aim of the research is to develop an understanding of a topic to inform health practice. Ethics approval for this study was obtained from the Victoria University of Wellington’s Human Ethics Committee (#30121) on 25 February 2022.

A qualitative design and a purposive snowball sampling approach was employed for this study. To explore a broad range of experiences of disabled people in accessing healthcare and support services during the pandemic, we undertook to select a sample that included variations in terms of the type of disability. In addition, participants were diverse in terms of ethnicity, age, and geographic spread around New Zealand. This diversity ensured that participants experience of accessing healthcare and disability support services captured the diversity, depth, and nuances of the research topic. This, in turn, further contributed to the credibility and validity of the findings. Participants were recruited by distributing invitations through organisations that supported disabled people, and also via Facebook forums and groups managed by disability advocacy organisations. Potential participants were asked to contact the research team directly via email. Interested individuals were sent information on the study and a consent form. To be included in the study, participants needed to speak English or be willing to work with a New Zealand Sign Language interpreter, and be either over 18 years of age and self-identify as disabled or be the parent or primary caregiver of a disabled child. They also needed to have interacted with the health and disability system during the pandemic.

Individuals participated in semi-structured interviews guided by an interview schedule between March–May 2022. During the interviews, participants were asked about their experiences in accessing healthcare and disability support services, what types of things helped or hindered their access, and the perceived short- and long-term impacts of the pandemic on their health and well-being. All participants provided written or verbal (if vision impaired) informed consent and permission for the interviews to be audio/video recorded. S.N.O. conducted the interviews remotely via Zoom. All interviews were with individual participants, except for two interviews with the Deaf community that, at the participants’ requests, were conducted as group interviews with a New Zealand Sign Language interpreter. Participants received a NZD 50 voucher to acknowledge their contribution to the research.

Interview recruitment ceased after interviews with 64 participants; these interviews covered a wide range of experiences related to healthcare and disability support and offered sufficient variation to identify where impairment and disability-specific variation occurred in experiences for Deaf and vision-impaired participants. Interviews lasted between 45 and 60 min, and participants could pause or stop the interview at any time. In total, 49 participants were disabled, and 15 interviewees were parents of disabled children who were under 18 years of age. Three participants brought support people to their interview. These individuals also consented and participated in the interviews.

All interviews were transcribed and deidentified, and participants were given the opportunity to review/comment on their transcripts. No participants requested any changes; each was assigned a unique number; parents were identified with a lowercase ‘p’ alongside this number (e.g., P3p), and situations where a participant also invited a support person to the interview were identified with a lowercase ‘s’. Participants came from a variety of backgrounds (Table 1) with 47 women, 14 men, and three people who identified as non-binary. The youngest participant was 18 years old, and the oldest participant was 73 years old, with most participants aged 30–39 years. Participants self-identified their impairments, with some participants identifying as living with multiple impairments.

### Analysis

In keeping with the underpinning methodology, a descriptive inductive approach was employed to explore participants’ experiences of accessing healthcare and disability support services, access barriers and facilitators, and short-term and long-term impacts of the pandemic on their health and well-being. During data collection, the research team met frequently to discuss emergent findings and the research direction, informing subsequent interviews. Notes were kept of these meetings, informing decision-making. Transcripts were thematically analysed through a process of careful reading and re-reading (firstly by S.N.O.), which resulted in the identification of emergent themes that focused on the barriers to accessing healthcare, impairment-specific impacts of the pandemic, mental wellbeing during the pandemic, and the short- and long-term impacts of the pandemic. These themes were then refined through a didactic and iterative writing approach amongst the research team, which resulted in three refined themes. With this analysis approach, researchers concentrate on comprehending the participant groups’ lived experiences in depth. This, in turn, facilitates the emergence of the meaning of events through a cycle of refining interpretations [17].

During analysis, researcher triangulation was employed to add rigor to our study and confirm data reliability. In this process, the research team discussed codes, emerging themes, and findings with each other. Potential errors, biases, or oversights were identified and removed from data, and the research team agreed on the final interpretation of data.

In addition, researcher diversity contributed to the triangulation process. The team had diverse backgrounds, areas of expertise, ethnicities, and ages; several are registered health professionals, and all have experience in disability research. Three members of the research team have disabilities, and two are primary caregivers of disabled children, which contributed to providing a lived experience lens to data analysis.

## 3. Result

Participants reported a broad range of experiences; analysis of interview transcripts resulted in three distinct themes that reflect their experiences: protective personal factors, the immediate pandemic policy impacts, and exacerbating factors. The themes are elaborated upon in the following sections.

### 3.1. Protective Personal Factors

A range of factors made accessing health and disability support services easier for disabled people during the pandemic, including maintaining positive relationships with service providers, possessing knowledge and skills to navigate the health and disability support system effectively, having access to individualised funding, and receiving support from family and whānau (i.e., the term for extended family group in the Māori language; this term is more complex than family as it captures flexible dynamics based on Indigenous Māori and tribal worldviews). In New Zealand, some disabled people have access to individualised funding, a type of financial support that allows disabled people to access government funding to organise and pay for the support and services they need. Unlike the traditional model of funding, where service providers receive funding and are contracted by the government to provide services, this funding model gives disabled people more choice and control over accessing disability support services:

*“It’s taken a long time to get it right. But it [Individualised Funding] works really well now. And life is so much easier having the right funding in place.”* (P59)

Individualised funding arrangements provide disabled people with more flexibility in accessing disability support services as participants are not forced to engage in a particular disability service; instead, they can organise and pay for support services of their choosing. This funding model proved particularly useful when the COVID-19 pandemic disrupted support service delivery, leaving those with individualised funding more options for accessing support; for instance, during lockdowns, when no support was available, individualised funding could be used to buy toys, learning materials, and outdoor equipment to assist disabled people and their families.

*“[For] the general day to day stuff that I can employ my own support staff, that we can be flexible with the funding on how that looks. That’s, that’s been really good.”* (P24)

Another protective factor that made it easier for disabled people to interact with the health and disability support systems was having the support of family and whānau. This access to physical and emotional support made a positive difference to disabled people. One participant explained that she would not have been able to access the healthcare that she needed without her mother attending.

*“I’m really glad that my mom came [to the appointment] because she was like, “this is really bad”. Because it was really inaccessible.”* (P10)

Having the support of family and whānau also assisted disabled people in coping with pandemic-related stress and the additional challenges it created for disabled people. One participant offered the following narrative that describes how, for her, family support meant she could cope.

*“To be honest, my family is like 80% [of] help to me… My father gets involved, my mum gets involved, my sister gets involved, her husband does. Their kids are nice to my child, they play with my child. Just so many things that go into a person’s wellbeing that if you don’t have family, I honestly would not be able to cope.”* (P62)

It is well recognised that the pandemic had a significant impact on the ability to maintain pre-pandemic levels of health and disability services [8,18]. For example, healthcare services were rationed in some places [9]. These impacts were felt by the participant group; however, having a good relationship with primary health service providers enabled disabled people to have better access to some services.

*“The only reason I think I had adequate healthcare is because I had GPs [general practitioners] that advocated for me. My GP, for instance, will keep calling the hospital and make sure I get a bed, I get treated, and they’ll keep ringing and ringing and ringing. My GP will try and follow up on everything so that I’ve got all the information in one place. If I was in hospital, she would ring the hospital and give them a rundown of everything.”* (P11)

Similarly, good relationships with disability service providers also facilitated access to support services. One participant described how a social worker supported her child’s access to hippotherapy (horse riding) during the pandemic, ultimately supporting her child’s wellbeing.

*“We are very lucky to have that [organisation name] disability social worker. She is amazing. She checks on us every other day. So even though she’s got a lot of clients, she still makes the time just to check in and see how we’re doing or if we need anything. She got [name] set up to go to RDA [Riding for the Disabled (RDA) refers to a number of organisations that aim to develop disabled people’s confidence, independence, and wellbeing through therapeutic horse-riding programmes)]. We got given a spot straightaway even though there’s a waitlist. She got us into RDA and other supports in place.”* (P13)

Besides maintaining good relationships with healthcare and disability support service providers, having health system literacy was another crucial factor in supporting health and wellbeing during the pandemic. One participant described how having the knowledge and skills to navigate the health and disability system was critical for disabled people to access services. She recognised the privilege that this knowledge afforded her.

*“I feel so lucky that I, I do have a really good understanding of how it [the system] works and how to best work within that system for the needs of my children.”* (P59)

Similarly, participants relayed that they had strategies to maximise the chances that they would be successful when approaching healthcare and disability services. Participants revealed they were intentionally tactical when approaching service providers to ensure they would be given access to services that supported their health and wellbeing. One strategy was understanding the government policies and guidelines to secure a positive response, as described by the following participant:

*“I find what guideline a person has to follow. And then I read that guideline. And then I use that guideline and the words that I need to use to get what I need, so they can’t turn me down.”* (P59)

In addition, to recognising the positive aspects of good relationships with care providers and how to navigate health and disability services, participants also discussed the barriers they encountered in some depth.

### 3.2. The Immediate Pandemic Policy Impacts

The COVID-19 pandemic response involved a range of policy and legislative changes to manage the public health ramifications of the virus. These measures created further barriers in the health and disability support system. These system-level barriers were related to changes to the healthcare and disability support environment such as infection prevention measures, and the added complexity of the healthcare and disability support system.

Access to services was negatively impacted by some COVID-19 policies that created a level of ‘caution’ in the health and disability support system. The caution altered the healthcare environment and led to disabled people feeling they were ‘de-humanised’ during interactions with health professionals. For instance, one participant was required to perform a sensitive and personally invasive examination on her disabled child because the doctor was following social distancing protocols.

*“They said come into the clinic, we went into the back door with the toilet, but it’s not a clinical room, it is right at the back door with all the rubbish and linen. He [the doctor] said pull down his pants and he just looked and said this is quite serious… if he’s in pain he needs an emergency surgery done. But luckily, I asked “are you in pain when I touch the swelling?” I did this instead of the doctor because he has a distance away… like he was very far away, I mean he’s right in the corner looking at us. We’re fully wearing the PPE [personal protective equipment]. [The doctor] did not touch nothing.”* (P28)

Social distancing and infection prevention measures resulted in various difficulties for disabled people in accessing health and disability services. One person described the challenges of not having support in completing paperwork, which impacted their wellbeing in the moment and added to the stress of attending such services.

*“When you go into a session… for mental health or whatever, you go through the form together in the office and talk through it, whereas now because of social distancing and all that the forms are being sent to me and I’m having to spend time reading them and filling them out and you know, it can be quite a strain on my eyesight, yeah, just not having so much interpersonal interaction is quite challenging and that’s all just to do with social distancing.”* (P25)

Many participants found enforced infection control measures problematic, especially when disabled people were not able to take a support person with them to healthcare services. This situation created unique challenges for disabled people and resulted in anxiety and trauma. One participant describes her experience of being admitted to hospital during the pandemic:

*“I wasn’t allowed any family in with me, so was my first time ever being in hospital alone. And it was very scary… I spent a very miserable three days in the hospital by myself… it was very anxious time and really put me off seeking hospital visits ever since really, I avoid it at any costs just because it… was emotionally scarring.”* (P25)

Pandemic restrictions created additional geographical barriers to access healthcare too. As a small country, some services are offered only in certain regions. During the pandemic, travel was not allowed to and from some regions, and as a result, people could not access their typical healthcare services. These travel restrictions prevented some participants from accessing the healthcare they needed.

*“I’ll go to Auckland four times a year… and I get four injections per day… I think I missed out on… six appointments over the past two years where I haven’t been able to go… because of the restrictions on travel because Auckland was in level three lockdown [One level below full lockdown (Alert Level 4), Level 3 was characterised by a medium risk of community transmission, active but managed infection clusters, and significant restrictions on travel, business, events, and gatherings.].”* (P57)

Like most national health systems, New Zealand’s health and disability system is complex; support and services are fragmented, planning and services vary, and the system lacks efficiency and consistency [4]. As a result, healthcare and disability support service consumers have highlighted that the system is difficult to navigate. The pandemic created additional layers in the system. For instance, services were fragmented, additional physical and communication barriers were put in place, eligibility assessment and extensive paperwork were introduced, and there was a lack of coordination. These pandemic-related changes made it more difficult for disabled people to find appropriate services, access and navigate them, and hence, leading to access issues for some participants:

*“The system is very complicated… navigating the healthcare system needs so much information that it’s actually really difficult. And a lot of the time you push it to one side, because it’s just too much to read, too much to go through, too much to work out.”* (P7)

In addition to navigating the healthcare system, disabled people struggled to gain accessible information about healthcare and disability support services. Information was not always available in appropriate formats such as large print, easy read, or with New Zealand Sign Language interpretation. In some cases, the information was available but was not communicated to disabled people by government agencies or healthcare providers. This meant that disabled people leveraged their social capital and networks to find out how to access the support they needed.

*“They don’t actually have any information for disabled people themselves. A lot of the conversations that I’ve had with friends who get support is us as friends supporting each other, trying to figure out plans for them, so that they can be kept safe but there’s no actual government guidance or any kind of assistance for that, so it feels very individualised.”* (P31)

Disabled people faced a range of challenges in their attempts to access various healthcare services, such as pharmacy, primary care, and hospital care. Widespread service cancellations and postponements meant many disabled people lost access to healthcare and support services. Perpetuating this, typical challenges around service accessibility and ableist attitudes continued to pose obstructions to accessing basic health and disability support. One participant described the impact of service delays on her child’s health in the following narrative.

*“I was worried because she basically went from doing something four to five days of a week to [nothing], physio and Botox all delayed as well, like you’d go every six months [for Botox injection] and then everything was delayed because of COVID.”* (P62)

During the pandemic, a range of healthcare services were altered to accommodate government public health mandates. As a result, healthcare was delivered in new and modified ways that accommodated social distancing requirements; however, this, combined with a lack of flexibility on the part of those delivering healthcare, led to additional impediments and reduced service accessibility for disabled people. One example of the reduced accessibility is offered in the following excerpt:

*“The majority of the blood test clinics had closed. And the ones that were still open, were entirely inaccessible in their physical setup for us. So, for example, I wanted to go get a blood test done for myself, but you can’t make appointments at the clinics. So we just had to turn up. And I asked where their mobility park was, and there wasn’t anywhere near the building. So ideally, if the mobility park had been close to the building, I would have been able to go there, park and leave [Name] in the car, go and get my blood test done. I asked if they’d be able to come out and do the blood tests while we were still in the car. They said they couldn’t… I ended up not getting that blood test done because it was just going to be too difficult.”* (P47)

In addition to disruption of access to health services, the pandemic disrupted disabled people’s access to disability support services too. This significantly impacted those disabled people who rely on regular care and support. In the following excerpt, a participant describes how she was unable to travel to get food and went hungry because of a loss of disability support services.

*“The biggest issue for me… was getting food, because I have a homecare nurse and when COVID landed that home care was completely taken away. I don’t live anywhere near the shops, I couldn’t get to the shops in any way at all. And I had no food and the attitude of the homecare person was well hard luck, then you’re gonna have to starve, aren’t you?”* (P38)

Furthermore, participants recognised that some service suspensions or significant disruptions, especially during level 3 and 4 lockdowns, were not correctly managed, and disabled people were unduly impacted by agencies or support workers misinterpreting policy changes. The following narrative highlights this point; however, participants were powerless to change the systems that led to the disruption.

*“They should still do my six hours when we’re in level 4 or 3 or whatever. They should supply the service that they’re supposed to supply when things are normal. But [in] level 3 or 4 they don’t supply at all. But they should do.”* (P29)

### 3.3. Exacerbating Factors: Compounding Vulnerabilities, Overstretched Systems, and Vaccine Mandate

Due to the pandemic, policy changes were made that worsened already limited access to healthcare and disability services for disabled individuals. This was further compounded by various factors, such as increased vulnerability among the disabled population, the overwhelming general demand on the healthcare and disability systems, the requirement for mandatory vaccinations and the impact on the disability support workforce, and the use of personal protective equipment.

Some participants had compounding risk factors besides impairment or disability that impacted their experiences of accessing healthcare during the pandemic. These factors can be better understood by considering the socioeconomic determinants of health. In addition to disability risk factors, participants also had to deal with multiple social identities with which they identified. One participant shared how the intersection of these identities affected their ability to obtain healthcare.

*“Being fat and also being a woman has definitely significantly influenced my experiences of healthcare, and how that plus being disabled, and also mentally ill, because I have anxiety and depression, how all of those things together can significantly influence someone’s ability to want to access healthcare in a pandemic, and their ability to access healthcare.”* (P6)

Prior to the pandemic, as with many health systems globally, health workforce shortages and increasing service demand stretched the New Zealand health and disability support system’s ability to respond to ‘business as usual’ situations. The COVID-19 pandemic exacerbated existing system pressures, and lockdowns and staff shortages resulted in longer than usual wait times. The health system struggled to provide sufficient services to meet community needs; there were delays and wait lists to access primary and secondary care, diagnostic tests, surgeries, and disability support services and equipment [19]. One participant described their experience in the following excerpt:

*“They were having like, less clinics, less everything. I wasn’t able to access the normal things in the health system that I need just to stay okay, day to day. I wasn’t able to access them.”* (P1)

Several participants spoke about early hospital discharge due to staff shortages. They felt clinicians were pressured to release patients quickly, particularly before weekends. A participant shared their experiences and provided a summary of the situation.

*“Obviously, there were all the COVID protocols, and people had been pulled off to do other things. And they were really behind with their surgery. So, you know, there was pressure on the health system… it certainly felt like there was a real kind of attempt to push people out without really being sure they were ready… that’s really quite unusual that you would kind of harass a patient to leave.”* (P55)

As part of the pandemic policy, the government mandated vaccination for health and disability workers. This created additional challenges for disabled people in accessing support from carers in a sector already facing significant staff shortages [12]. One example of this was that the vaccine mandates (COVID-19 Public Health Response (Vaccinations) Order 2021 [20]) specifically prevented disabled people with individualised funding from hiring unvaccinated carers (all vaccination mandates for health and disability workers ended on Sunday, 26 September 2022). Although some disabled people appreciated being protected from the virus, this mandate eliminated the choice to employ support workers and created specific challenges, as one participant highlighted in the following narrative.

*“I’ve been wanting to hire support workers for various things like learning to cook and practising cooking and stuff like that. And I’ve been wanting to hire them, but all three of them happen to be unvaccinated and… because of the government policy… I can’t [hire people] with the Government funding because it’s illegal for me to do so because they are unvaccinated, and still to this day that policy exists. So, I still can’t hire them. And I’ve been waiting… months and months now, and some of my funding might end up expiring because I haven’t been able to get them all this time.”* (P39)

While vaccination was a valuable public health measure during the pandemic, mandatory vaccination orders created obstacles for disabled people who were not eligible for COVID-19 vaccination or for those disabled people who chose not to get the vaccine. This group of disabled people faced challenges in accessing a range of healthcare and support services. The following participant described one such situation where their son was refused healthcare because they (the parents) could not be vaccinated, and the parent was not yet eligible for vaccination due to staggered roll out of vaccination for different population groups:

*“They said in order to complete testing, first, my son and I had to be fully vaccinated in order to go into the clinic because of the concerns about COVID. [Name] and I needed to both be fully vaccinated as a requirement of getting access to their services.”* (P55)

In addition to vaccinations creating obstacles, the legislated and mandatory use of personal protective equipment resulted in challenges. Some disabled people, if eligible due to health reasons, could apply for and receive a mask-wearing exemption from the Ministry of Health. While this group of disabled people did not need to wear a mask in public spaces, participants described situations where, despite being approved for a mask exemption, they were unable to access healthcare and disability support services:

*“In [main centre]… you are not allowed to go in this health building… you are banned if you don’t wear a mask, including if you have a mask exemption.”* (P39)

Mandatory mask wearing created significant challenges for Deaf people who used lip-reading for communication. One Deaf participant described how this requirement caused her significant anxiety, and as a result she chose to avoid primary healthcare.

*“Through COVID, for two years, it would stress me out going there [GP practice] because of the mask situation and there were just too many rules to follow.”* (P42)

Pandemic-related healthcare challenges were so significant for the Deaf community and vision-impaired participants that the findings have been analysed in two separate articles (see Roguski et al. [13] and Good et al. [11], respectively). Deaf participants raised a myriad of issues around accessing healthcare, which led many to opt out of accessing it except in emergency situations.

## 4. Discussion

Our findings affirm that disabled New Zealanders’ access to healthcare and disability support services were negatively impacted by the pandemic, consistent with extant literature [8,11,13,21,22,23]. Our study has revealed that, while policy decisions generally negatively impacted the healthcare and disability support of disabled individuals, some policies resulted in positive outcomes for disabled people. For example, there was an increase in accessible communication such as sign language for public health announcements and easy read materials. There were also some financial assistance programmes implemented to assist disabled people. More specifically, disabled people with individualised funding spoke of the benefit of this during the pandemic when accessing healthcare and disability support services. However, most pandemic policies had unintended effects on disabled people, in part due to increased healthcare demands that overwhelmed an already overstretched healthcare and disability support system. In contrast, participants emphasised the impact proactive health professional action and advocacy had on their healthcare trajectories. Healthcare professionals, at times, played a significant role by advocating for disabled people and ensuring relevant information was communicated and access and services were coordinated. Such findings mirror those overseas and nationally where having strong, established relationships positively influences service outcomes [18,24,25].

Disabled individuals have faced difficulty accessing various services due to a multitude of reasons, including physical and spatial barriers, inaccessible digital platforms, and inadequate education and awareness among service providers regarding disabilities. To provide disabled people with accessible services, their diverse accessibility needs should be considered. To enhance accessibility for disabled people, healthcare professionals require disability accessibility training, so they understand those aspects of services that need to be tailored to disabled people. From a policy perspective, requirements for disability-specific training should be embedded in codes of practice and competency standards.

Participants emphasised the crucial role support people play when receiving healthcare services. Pandemic-mandated changes to health service delivery meant that support people (both formal, such as interpreters, and informal, such as family members) were not allowed to attend appointments. The inclusion of support people to aid communication and accessibility is a fundamental human right and a requirement frequently overlooked by the health system [26,27]. Future pandemic policy requirements should recognise this right for disabled people and enable the inclusion of appropriate support people at every healthcare access interaction. A starting place for implementing this could be through mandating healthcare professionals complete regular training around the New Zealand code of rights, and have a general understanding of the impact of good health service delivery on meeting human rights, to ensure that services meet a minimum standard.

Our research highlights the inadequacies of the public health systems in information sharing in support of disabled people during the pandemic. These findings mirror those in the literature confirming a lack of utilisation of existing disability support networks and inclusive communications and information to support disabled people during the COVID-19 pandemic [28]. Future policy direction should consider the ability to mobilise existing networks within the disabled community, as these have been demonstrated to be highly effective in supporting the disabled community during the pandemic [28]. Pragmatically, this sort of mobilisation should work in conjunction with public health professionals to provide evidence-informed directives. To rectify these inadequacies, healthcare and disability support systems need to recognise the importance of disability support networks and acknowledge their role in ensuring disabled people receive, find, and access the information and support they need. A first step in ensuring this could include maintaining and auditing records around the activities of different networks to ensure the healthcare and disability support system can easily identify core parties that are able to be mobilised in cases of future emergency management.

To ensure disabled people have better access to healthcare in future pandemics, their experiences and views need to be captured, understood, learned from, and built into planning and preparedness; ideas that are also supported by Seth and colleagues [29]. Disabled people and their representative organisations must be at the centre of planning, and contribute to the guidelines, service delivery arrangements, and service continuity plans. Our study strongly emphasises the need to consider how policy is implemented alongside its intent. For example, while mandatory vaccinations of support workers may be effective in managing COVID-19 transmission, without considering how to ensure a supply of vaccinated support workers, adverse effects ensue. Such effects have been reported elsewhere [30,31]. In addition, there needs to be a dedicated plan and response that covers the specific needs and wants of disabled people, their culture, language needs, and required support. A first step in bringing a disability-centric view to planning, therefore, should begin with representative organisations to undertake a mapping exercise to identify all unintended consequences of COVID-19 policy decisions.

Further learning is required about how previous negative healthcare experiences influence people’s perception of and ability to try to access health and disability services [32]. Other research suggests that these experiences may lead populations to access healthcare later when their care needs are greater [33,34]. Given the high use disabled people make of healthcare services, there remains a greater unmet need for health services post-pandemic, and perhaps more concerningly, worse health outcomes delivered at higher cost. It is pertinent that policymakers continue to review and evaluate disabled people’s ability to access timely healthcare.

Additional research is also required to understand the impact of COVID-19-related policy changes on the long-term functioning of the health and disability support system, this could include analysis of quantitative data on hospital admissions and service waiting times. As a qualitative study, findings are not necessarily generalisable to all disabled people; however, the breadth of disabilities covered in this study means the findings may be transferred with care across similar populations. It is particularly noteworthy that, given the diversity in participants, both from a disability perspective, and from general demographic differences in age and gender, the research strongly identified three core themes applying across the population. Future research should consider the views of disabled older populations, including those living in residential aged care facilities; while our research has captured the views of a wide population, the reliance on social media channels means that we may have omitted a population with particularly high healthcare and disability support service access needs. One core step to managing future health system requirements might be facilitating co-design workshops between health and disability support service users, clinicians, and policymakers around pandemic planning for the disabled population.

## 5. Conclusions

The COVID-19 pandemic significantly increased the demand for healthcare and disability support services in an already stretched healthcare and disability support system, disrupting disabled people’s access to services. This study revealed three themes that influence disabled people’s health service journeys. These relate to personal protective factors, the immediate increase in system complexity caused by policy changes, and the implications of policy change in those with exacerbating factors, including those with compounding vulnerabilities. Research findings revealed the significant impact of service disruption on disabled people. This research demonstrates that disabled populations must be central to future health and disability support planning. This includes involvement in the preparation, design, implementation, and planning of future healthcare services and disability support systems to ensure accessible service continuity.

## Figures and Tables

**Table 1:** Participant demographics.

Participant	Gender	Self-Reported Impairment(s) of Participant/Disabled Child	Age Range (Years)	Ethnicity
1	Female	Mental illness Mobility	40–49	NZ European
2	Female	Vision impairment	30–39	NZ European
3p	Female	Autism with developmental delay	30–39	Māori NZ European
4	Female	Vision impairment	30–39	NZ European
5	Male	Mobility	30–39	NZ European
6	Female	Anxiety Chronic depression Fibromyalgia	18–29	NZ European
7	Female	Mobility	50–59	NZ European
8	Female	Mobility	40–49	NZ European
9	Male	Attention deficit hyperactivity disorder (ADHD) Dyslexia Vision impairment	18–29	Māori NZ European
10	Non-binary	Anxiety Depression Dyslexia Vision impairment	18–29	NZ European
11	Female	Auto-immune disease Mobility	30–39	Indian
12	Female	Cerebral palsy Mobility	60+	NZ European
13p	Female	ADHD Asthma Autism Intellectual disability	30–39	Māori NZ European
14	Male	Vision impairment	18–29	NZ European
15	Male	Autism Antisocial personality disorder	18–29	NZ European
16	Female	Cerebral palsy	50–59	NZ European
17p	Female	Autism with global developmental delay	40–49	Māori NZ European
18p	Female	Autism	40–49	Māori NZ European
19s	Male	Autism Global developmental delay	18–29	Māori NZ European
20p	Female	Cerebral palsy Intellectual disability	30–39	Chinese
21	Male	Mobility Muscular dystrophy	60+	NZ European
22	Female	Vision impairment	40–49	NZ European
23	Female	Mobility	60+	NZ European
24s	Female	Down syndrome	18–29	NZ European
25	Female	Myalgic encephalomyelitis	18–29	NZ European
26	Female	Cerebral palsy	30–39	NZ European
27p	Female	Autism	30–39	Irish
28p	Female	ADHD Autism	30–39	Malaysian
29	Male	Vision impairment	60+	Māori NZ European
30	Female	ADHD Ehlers-Danlos Syndrome Mobility	40–49	New Zealand European
31	Non-binary	Cerebral palsy	30–39	South African
32	Male	Mobility	50–59	Indian
33p	Female	Autism Intellectual disability	40–49	Māori NZ European
34	Male	Cerebral palsy	40–49	NZ European
35	Female	Mobility	50–59	NZ European
36	Female	Vision impairment	50–59	South African
37s	Male	Autism Learning disability	30–39	NZ European
38	Female	Vision impairment	60+	NZ European
39	Male	Autism Asperger’s syndrome	18–29	Swiss English
40	Female	Down syndrome	30–39	NZ European
41	Non-binary	Hard of hearing Mobility	60+	NZ European
42	Female	Deaf	40–49	NZ European
43	Female	Deaf	50–59	NZ European South African
44	Female	Deaf	50–59	South African
45	Female	Autism	18–29	NZ European
46	Female	Mobility	50–59	NZ European
47p	Female	Down syndrome Learning disabilities	30–39	NZ European
48	Female	Deaf	50–59	NZ European
49	Female	Deaf	30–39	NZ European
50	Male	Deaf	50–59	NZ European
51	Female	Deaf	40–49	NZ European
52	Male	Deaf	30–39	NZ European
53	Female	Deaf	40–49	Filipino
54	Female	Deaf	30–39	NZ European
55	Female	Fistulising Crohn’s disease	40–49	NZ European
56p	Female	Cerebral palsy Epilepsy Non-ambulatory Non-verbal Sensory processing issues	30–39	Māori
57	Female	Ehlers-Danlos Syndrome Mastocytosis	18–29	NZ European
58	Female	Parkinson’s disease Loss of sight	60+	NZ European
59p	Female	Autism	30–39	Māori NZ European
60	Male	Cerebral palsy	50–59	NZ European
61p	Female	Autism	30–39	Māori NZ European
62p	Female	Cerebral palsy	30–39	NZ European
63p	Female	Down syndrome	30–39	NZ European
64p	Female	Autism Down syndrome	40–49	NZ European

## Data Availability

Data are contained within the article.
